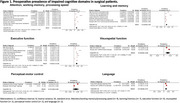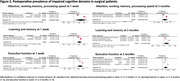# What is the prevalence of impaired cognitive domains in surgical patients? A systematic review and meta‐analysis

**DOI:** 10.1002/alz70857_099172

**Published:** 2025-12-24

**Authors:** Subin Park, Ellene Yan, Ray Martinez‐Rodriguez, Bijal Desai, Jonathan Chung, Aparna Saripella, Marina Englesakis, Keera N. Fishman, Frances Chung

**Affiliations:** ^1^ University of Toronto, Toronto, ON, Canada; ^2^ Toronto Western Hospital, University Health Network, Toronto, ON, Canada; ^3^ Temerty Faculty of Medicine, University of Toronto, Toronto, ON, Canada; ^4^ University Health Network, Toronto, ON, Canada; ^5^ Baycrest Hospital, Toronto, ON, Canada; ^6^ Ontario Shores Centre for Mental Health Sciences, Toronto, ON, Canada

## Abstract

**Background:**

Cognitive impairment is prevalent in the surgical population but remains significantly under‐recognized. This systematic review and meta‐analysis aims to (1) assess the prevalence of specific impaired cognitive domains in surgical patients perioperatively and (2) examine postoperative changes across the domains.

**Methods:**

A comprehensive search was conducted in five electronic databases from inception until March 19, 2024. Studies were included if they met the following criteria: (1) surgical patients ≥18 years of age; (2) sample size of ≥100 surgical patients; (3) assessed cognitive domain(s) preoperatively with a neuropsychological battery or clinical evaluation; and (4) reported the prevalence of impaired cognitive domains or changes perioperatively. The exclusion criteria included neurosurgery; those with overlapping data; cross‐sectional, case control, and case series studies; and non‐English articles.

**Results:**

Of the 12,082 articles screened, 21 studies with 5,725 patients were included (11 non‐cardiac surgery, 10 cardiac surgery). Preoperatively, executive function had the highest pooled prevalence of impairment (18%; 95% CI: 13%, 24%), followed by visuospatial function (16%; 95% CI: 6%, 26%) and attention/working memory/processing speed (14%, 95% CI: 9%, 18%). Rates were lower in perceptual‐motor control (13%; 95% CI: 9%, 36%), language (13%; 95% CI: 8%, 17%), and learning/memory (12%; 95% CI: 8%, 16%) (Figure 1). Postoperatively, impairment was most pronounced at one week, with 35% (95% CI: 4%, 66%) in attention/working memory/processing speed, 34% (95% CI: 16%, 51%) in executive function, and 28% (95% CI: 16%, 40%) in learning/memory. Impairment reduced at three months postoperatively, with a prevalence of 16% (95% CI: 3%, 35%) in attention/working memory/processing speed, 15% (95% CI: 6%, 24%) in executive function, and 12% (95% CI: ‐2%, 25%) in learning/memory (Figure 2).

**Conclusions:**

Cognitive domains were impaired preoperatively in 12% to 18% of surgical patients. The most commonly impaired domains were executive function, visuospatial function, and attention/working memory/processing speed. One‐week post‐surgery, the prevalence of impaired cognitive domains rose to 28% to 35%, then decreased by three months to levels similar to preoperative rates (12% to 16%). Identifying commonly impaired cognitive domains can inform the selection of cognitive screening tools to assess surgical patients at greater risk for adverse outcomes.